# Different antibody-associated autoimmune diseases have distinct patterns of T follicular cell dysregulation

**DOI:** 10.1038/s41598-022-21576-8

**Published:** 2022-10-21

**Authors:** Filipa Ribeiro, Vasco C. Romão, Sara Rosa, Kátia Jesus, Ana Água-Doce, Sofia C. Barreira, Patrícia Martins, Susana Lopes da Silva, Ema Nobre, Maria João Bugalho, Válter R. Fonseca, João Eurico Fonseca, Luis Graca

**Affiliations:** 1 Instituto de Medicina Molecular João Lobo Antunes, Faculdade de Medicina da Universidade de Lisboa, Lisbon Academic Medical Centre, Avenida Professor Egas Moniz, 1649-028 Lisboa, Portugal; 2 Instituto Gulbenkian de Ciência, Oeiras, Portugal; 3 Rheumatology Department, Hospital de Santa Maria, Centro Hospitalar Lisboa Norte, Lisbon Academic Medical Centre, Lisboa, Portugal

**Keywords:** Autoimmunity, T cells

## Abstract

Autoantibodies are produced within germinal centers (GC), in a process regulated by interactions between B, T follicular helper (Tfh), and T follicular regulatory (Tfr) cells. The GC dysregulation in human autoimmunity has been inferred from circulating cells, albeit with conflicting results due to diverse experimental approaches. We applied a consistent approach to compare circulating Tfr and Tfh subsets in patients with different autoimmune diseases. We recruited 97 participants, including 72 patients with Hashimoto’s thyroiditis (HT, n = 18), rheumatoid arthritis (RA, n = 16), or systemic lupus erythematosus (SLE, n = 32), and 31 matched healthy donors (HD). We found that the frequency of circulating T follicular subsets differed across diseases. Patients with HT had an increased frequency of blood Tfh cells (*p* = 0.0215) and a reduced Tfr/Tfh ratio (*p* = 0.0338) when compared with HD. This was not observed in patients with systemic autoimmune rheumatic diseases (RA, SLE), who had a reduction in both Tfh (*p* = 0.0494 and *p* = 0.0392, respectively) and Tfr (*p* = 0.0003 and *p* = 0.0001, respectively) cells, resulting in an unchanged Tfr/Tfh ratio. Activated PD-1^+^ICOS^+^Tfh and CD4^+^PD-1^+^CXCR5^–^Tph cells were raised only in patients with SLE (*p* = 0.0022 and *p* = 0.0054), without association with disease activity. Our data suggest that GC dysregulation, assessed by T follicular subsets, is not uniform in human autoimmunity. Specific patterns of dysregulation may become potential biomarkers for disease and patient stratification.

## Introduction

The finding that T follicular helper (Tfh) and T follicular regulatory (Tfr) cells control GC reactions prompted the study of circulating T follicular subsets in human autoimmunity^1–4^. Tfr cells derive from Foxp3^+^ Treg cells^5–7^, have a TCR repertoire directed to self-antigens and distinct from the repertoire of Tfh cells^8^, have a suppressive function^9,10^, and are essential to prevent the generation of autoimmunity in mice^11–13^. It was, therefore, tempting to speculate that a dysregulated Tfr/Tfh ratio could be implicated in antibody-mediated autoimmunity. We previously demonstrated that patients with primary Sjögren’s syndrome (pSS) had an increase in circulating Tfr cells compared to healthy donors (HD)^4,14^. Moreover, a subset of pSS patients with more autoantibodies and ectopic lymphoid structures (ELS) within minor salivary glands had a significantly higher Tfr/Tfh ratio^14^. We demonstrated that the generation of circulating Tfr cells is a normal process following a humoral response, namely influenza vaccination^4^, and can explain the high Tfr/Tfh ratio following chronic inflammatory reactions, as in pSS^4,14^. Although other researchers subsequently confirmed our results^15^, there were also conflicting reports^16^. Furthermore, reports have also been inconsistent in other autoimmune diseases, suggesting an increase or decrease of the Tfr/Tfh ratio^14,17–19^.

It has been difficult to compare those studies as the experimental approaches are not directly comparable. For instance, we previously found that circulating Tfr cells have an immature phenotype, given the developmental bifurcation of Tfr cells within human lymphoid tissue^4,20^. However, in some studies, Tfr cells are selected within CD45RO^+^ or PD-1^+^ cells^17,21^, excluding a large proportion of this cell population. CD25 may also be a poor marker for Tfr cells, especially the most mature within the GC, although circulating Tfr cells are CD25^+^^20,22–25^. Conversely, Tfh cells are often studied without exclusion of Foxp3^+^ cells, or selecting them as PD-1^+^, thus including both T follicular subsets (Tfh and Tfr) as Tfh cells and/or excluding immature PD-1^–^ Tfh cells^26,27^.

Given our results showing that pSS patients with ELS had a high Tfr/Tfh ratio, we addressed the hypothesis that other autoimmune diseases with target organ involvement, autoantibodies’ production and ELS would have a similar profile. For that purpose, we studied patients with Hashimoto’s thyroiditis (HT), an organ-specific autoimmune disease with ELS formation in thyroid gland. In addition, we aimed to investigate whether these findings could be replicated in other, more systemic, autoimmune diseases. Thus, using a consistent approach, we included patients with two different autoimmune rheumatic diseases: rheumatoid arthritis (RA), a systemic disease with a clear-cut target (i.e., articular) involvement; and systemic lupus erythematosus (SLE), the prototypical example of an autoantibody-driven systemic autoimmune rheumatic disease.

## Patients and methods

### Human samples

We recruited patients with HT (n = 18) followed in the Endocrinology Department of Hospital de Santa Maria, Centro Hospitalar Universitário Lisboa Norte (CHULN), Lisbon Academic Medical Centre, Lisbon, Portugal. Further, we included patients with RA (n = 16) and SLE (n = 32), who were followed in the Rheumatology Department of the same hospital. Only adult, non-pregnant, patients fulfilling the clinical classification criteria for HT (hypothyroidism with the presence of TPO antibodies), RA (American College of Rheumatology (ACR)/European Alliance of Associations for Rheumatology (EULAR) 2010), or SLE (ACR 1997) were included. We excluded patients under immunosuppressive treatment other than methotrexate, hydroxychloroquine, or ≤ 7.5 mg per day of prednisolone or equivalent. Patients diagnosed as having an infectious disease or diabetes, or who had received any vaccine in the previous month were also excluded. Disease activity was evaluated using the Disease Activity Score-28 (DAS-28) for RA patients, and Systemic Lupus Erythematosus Disease Activity Index (SLEDAI) for SLE patients. Relevant autoantibodies and serological markers of disease/disease activity were also collected. Fresh blood samples were collected in EDTA-coated tubes and processed on the same day for flow cytometry analysis. Finally, we collected fresh blood samples from 31 sex- and age-matched healthy volunteers.

### Ethical approval

This study was approved by the Lisbon Academic Medical Centre Ethics Committee (reference no. 505/14). Informed consent was obtained from all participants. All methods were performed in accordance with relevant guidelines and regulations.

### Cell isolation and flow cytometry

Peripheral blood mononuclear cells (PBMCs) were isolated by Ficoll gradient medium (Histopaque-1077; Sigma-Aldrich) using SepMate tubes (Stem-Cell Technologies). Cells were stained with anti-CD4 (OKT4, BioLegend), anti-CD45RO (UCHL1, BioLegend), anti-CD25 (BC96, eBioscience), anti-CD185/CXCR5 (J252D4, BioLegend), anti-CD279/PD-1 (EH12.2H7, BioLegend), anti-CD278/ICOS (C398.4A, BioLegend), anti-CCR6 (G034E3, BioLegend), anti-CXCR3 (G025H7, BioLegend) and anti-Foxp3 (PCH101, eBioscience). Intracellular Foxp3 staining was performed using the Foxp3 Fix/Perm Kit (eBioscience) according to the manufacturer’s instructions. Samples were acquired on a BD LSRFortessa cytometer (BD Biosciences) and further analyzed on FlowJo software (TreeStar).

### Statistical analysis

The normality of data was evaluated using the Shapiro–Wilk normality test. Kruskal–Wallis one-way analysis of variance (ANOVA) test with Dunn’s comparison post-test was applied for pairwise multiple comparisons, and two-way ANOVA with Dunnett’s test was performed for multiple comparisons. *P* values less than 0.05 were considered significant. Statistical and graphic analyzes were performed using GraphPad Prism software.

## Results

### The circulating Tfr/Tfh ratio is heterogeneous among distinct autoimmune diseases

**Figure 1:**
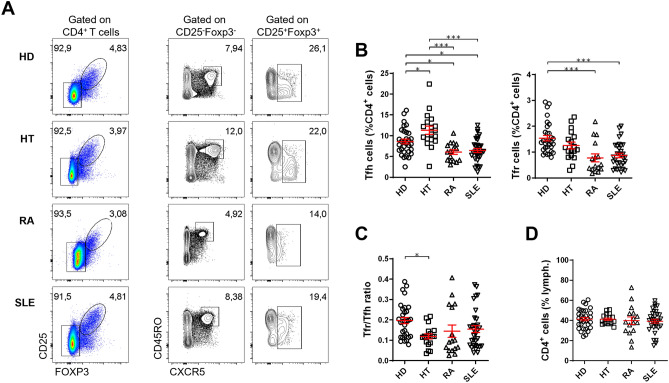
The circulating Tfr/Tfh ratio is heterogeneous among different autoimmune diseases. **(A)** Representative plots and **(B)** pooled data of the frequency of CD4^+^CXCR5^+^CD25^–^Foxp3^–^ Tfh cells and CD4^+^CXCR5^+^CD25^+^Foxp3^+^ Tfr cells in peripheral blood of patients with Hashimoto’s thyroiditis (HT, n = 18), rheumatoid arthritis (RA, n = 16), systemic lupus erythematosus (SLE, n = 32), and sex- and age-matched healthy donors (HD, n = 31). **(C)** Blood Tfr/Tfh ratio and (**D**) frequency of CD4^+^ T cells in HT, RA, SLE, and sex- and age-matched HD. Each data point represents one individual subject; bars represent mean ± SEM; *p < 0.05, **p < 0.01, ***p < 0.001, Kruskal–Wallis one-way analysis of variance (ANOVA) test with Dunn’s comparison post-test.

One of the major challenges in comparing studies of circulating T follicular cells in autoimmune diseases is the inconsistency of the approaches applied to select these cells by flow cytometry, namely the disparity in the markers used. To maintain a consistent strategy to analyze T follicular subsets by flow cytometry, allowing comparability among diseases, we investigated the profile of circulating Tfh cells (defined as CD4^+^CXCR5^+^CD45RO^+^CD25^–^Foxp3^–^) and Tfr cells (defined as CD4^+^CXCR5^+^CD25^+^ Foxp3^+^) in distinct antibody-mediated autoimmune diseases (Fig. 1A). We included patients with HT, representing an organ-specific autoimmune disease; RA, an autoimmune rheumatic disease with target involvement; and SLE, the prototypical autoantibody-driven systemic autoimmune disease (Table 1). All patients with HT had hypothyroidism with the presence of thyroid-specific autoantibodies, and the majority were treated with levothyroxine. Patients with RA and SLE were in line with the nature of an established disease cohort, with low disease activity and an expected frequency of autoantibodies.

**Table 1:** Demographic and clinical characteristics of study participants.

	HD (n = 31)	HT (n = 18)	RA (n = 16)	SLE (n = 32)
Age, mean ± SD years*	48.6 ± 17.4	49.0 ± 14.0	53.9 ± 15.7	52.8 ± 16.6
Female, no. (%)*	26 (81)	17 (94)	14 (88)	26 (81)
Disease duration, mean ± SD years	–	7.8 ± 12.7	12.1 ± 10.1	12.3 ± 8.4
DAS28, mean ± SD	–	–	2.9 ± 1.4	–
SLEDAI, mean ± SD	–	–	–	1.8 ± 1.8
Anti-TG positive (> 40 U/mL), no. (%)**		12 (75)		
Anti-TPO positive (> 60 U/mL, no. (%)**	–	14 (88)	–	–
Anti-CCP positive, no. (%)	–	–	13 (81)	–
RF positive, no. (%)	–	–	15 (94)	–
ANA ≥ 1/160, no. (%)	–	–	–	26 (81)
Anti-SSA positive, no. (%)	–	–	–	6 (19)
Anti-SSB positive, no. (%)	–	–	–	2 (6)
Anti-Sm positive, no. (%)	–	–	–	5 (16)
Anti-RNP positive, no. (%)	–	–	–	9 (28)
Anti-dsDNA positive, no. (%)	–	–	–	14 (44)
Methotrexate, no. (%)	–	–	17 (100)	4 (13)
Prednisolone, no. (%)	–	–	12 (75)	25 (78)
Hydroxychloroquine, no. (%)	–	–	5 (31)	29 (91)
Sulfasalazine, no. (%)	–	–	4 (25)	–
Azathioprine, no. (%)	–	–	–	6 (19)
Levothyroxin, no. (%)	–	17 (94)	–	–

*HD* healthy donors, *HT* hashimoto’s thyroiditis, *RA* rheumatoid arthritis, *SLE* systemic lupus erythematosus, *DAS28* disease activity score-28, *SLEDAI* systemic lupus erythematosus disease activity index, *Anti-TG* anti-thyroglobulin antibodies, *Anti-TPO* anti-thyroid peroxidase antibodies, *Anti-CCP* anti-cyclic citrullinated peptides, *RF* rheumatoid factor, *ANA* antinuclear antibody.

*There were no significant differences against HD controls.

**Anti-TG and anti-TPO titers unavailable for 2 patients.

Surprisingly, the frequency of Tfh and Tfr cells within the CD4^+^ T cell population varied substantially among the different diseases (Fig. 1A,B). Patients with HT showed an increased frequency of Tfh cells compared to HD (*p* = 0.0215). In contrast, the frequency of circulating Tfh cells was decreased in patients with RA and SLE (*p* = 0.0494 and *p* = 0.0392, respectively). Furthermore, RA and SLE patients also displayed reduced frequency of Tfr cells (*p* = 0.0003 and *p* = 0.0001, respectively), which was not observed in HT patients. Similar results were observed with the absolute cell numbers (Supplementary Fig. S1). These findings contrast with our previous results in patients with pSS, who had similar Tfh and higher Tfr frequencies than HD^14^.

Contrary to what we had hypothesized, patients with HT had a significant reduction in Tfr/Tfh ratio (*p* = 0.0338) (Fig. 1C), rather than an increase, as reported for pSS, an autoimmune disease which also has predominant target-organ (i.e., salivary/lacrimal gland) involvement^14^. Of note, the low Tfr/Tfh ratio in HT patients was mainly driven by an increase in the frequency of blood Tfh cells (Fig. 1A–C), which were previously shown to be unchanged in patients with pSS^14^. No correlation was observed between the frequency and the number of Tfh cells with the serum antibody titers of HT patients (Supplementary Fig. S2). Furthermore, the reductions in circulating Tfh and Tfr cells observed in RA and SLE resulted in Tfr/Tfh ratios similar to healthy controls. The results concerning RA are overall in accordance with what we have previously demonstrated for patients with established RA in remission/low disease activity^28^.

Notably, the Tfr/Tfh ratio from RA and SLE patients showed a significant interindividual variation (Fig. 1C). We could not find any clinical parameters, such as disease duration, disease activity, autoantibody status, or treatment that could explain the interindividual variability of the Tfr/Tfh ratio among these patients (Supplementary Figs. S3 and S4). Nevertheless, the Tfr/Tfh ratio in patients with RA in remission (DAS28 ≤ 2.6) tends to be higher when compared to patients who are not in remission, as previously reported^28^. As anticipated, we did not find significant differences regarding the frequency of CD4^+^ T cells (Fig. 1D).

Overall, these results show that different antibody-mediated autoimmune diseases have distinct patterns of circulating Tfh and Tfr cells involved in the regulation of GCs, suggesting that these diseases may have distinct forms of GC dysregulation.

### Activated PD-1^+^ICOS^+^ Tfh cells are increased in some, but not all, autoimmune diseases

**Figure 2:**
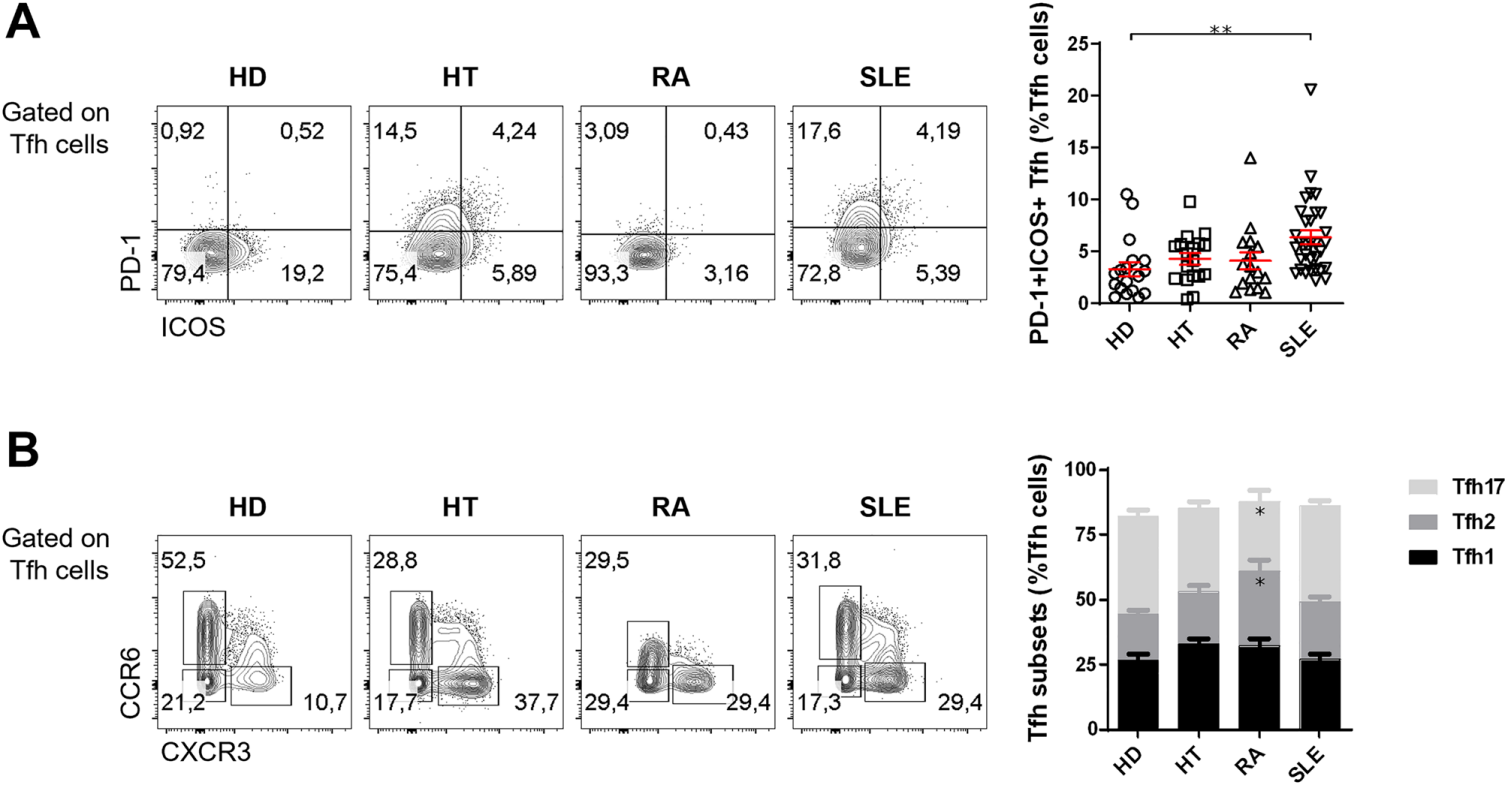
Activated PD-1^+^ICOS^+^ Tfh cells are expanded in SLE patients. **(A)** Representative plots (left) and pooled data (right) of the frequency of activated PD-1^+^ICOS^+^ Tfh cells in the peripheral blood of HT (n = 18), RA (n = 16), and SLE (n = 32) patients, and sex- and age-matched HD (n = 18). Each data point represents an individual subject; bars represent mean ± SEM; *p < 0.05, **p < 0.01, ***p < 0.001, Kruskal–Wallis one-way analysis of variance (ANOVA) test with Dunn’s comparison post-test **(B)** Representative plots (left) and pooled data (right) of the distribution of Th1-like (CXCR3^+^CCR6^–^), Th2-like (CXCR3^–^CCR6^–^), and Th17-like (CXCR3^–^CCR6^+^) Tfh cell subsets in HT (n = 18), RA (n = 16), and SLE (n = 32) patients, and sex- and age-matched HD (n = 19). *p < 0.05, **p < 0.01, ***p < 0.001, two-way ANOVA with Dunnett’s multiple comparisons test was performed.

After assessing the overall Tfh and Tfr populations, we investigated the frequency of Tfh subsets. First, we quantified the frequency of activated Tfh cells, defined as PD-1^+^ICOS^+^ cells within the Tfh gate (Fig. 2A). The PD-1^+^ICOS^+^ cells represent the population of Tfh cells that transiently increases in circulation shortly after immune stimulation, such as that provided by influenza vaccination^29–31^. We found a trend for a higher frequency of PD-1^+^ICOS^+^ Tfh cells in patients with organ-specific and systemic autoimmune disease, in comparison to healthy controls (Fig. 2A and Supplementary Fig. S1). This increase was, however, statistically significant only for patients with SLE (*p* = 0.0022) (Fig. 2A and Supplementary Fig. S1). No correlation between PD-1^+^ICOS^+^ Tfh cells of RA and SLE patients with disease activity was observed, possibly due to the low number of patients with high disease activity, as these are established disease cohorts (Supplementary Fig. S5). Moreover, no correlation of disease activity with total Tfh cells, both in number and percentage, was also observed (Supplementary Fig. S5).

It has also been reported that circulating Tfh cells can be subsetted in Th1-like, Th2-like, and Th17-like Tfh cells based on CCR6 and CXCR3 expression^2^. We did not find significant differences regarding the frequency of Tfh1, Tfh2, and Tfh17 subsets in patients with HT or SLE, compared to HD. However, we observed a decrease of Tfh17 cells in RA patients (*p* = 0.0109), with a converse increase of the Tfh2 cells (*p* = 0.0105) (Fig. 2B).

### Circulating CD4^+^PD-1^+^CXCR5^–^ Tph cells and CXCR5^+^CD25^–^Foxp3^+^ T follicular cells are increased in patients with SLE

**Figure 3:**
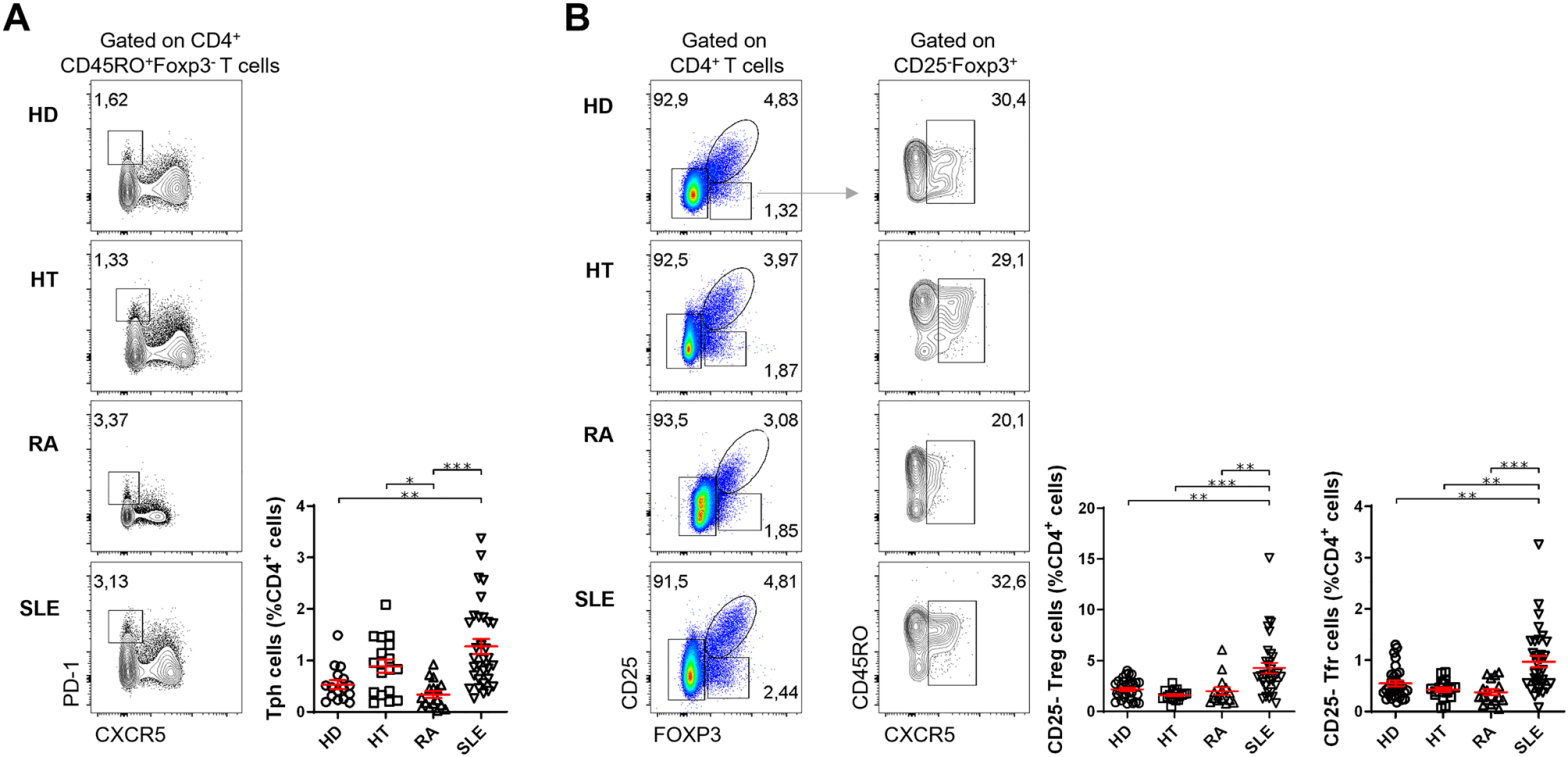
SLE patients show an increased frequency of circulating Tph and CXCR5^+^CD25^–^Foxp3^+^ T follicular cells. Representative plots (left) and pooled data (right) of the frequency of **(A)** Tph cells and **(B)** CXCR5^+^CD25^–^Foxp3^+^ T cells in the peripheral blood of HT (n = 18), RA (n = 16), and SLE (n = 32) patients, and sex- and age-matched HD (A: n = 16; B: n = 31). Each data point represents an individual subject; bars represent mean ± SEM; *p < 0.05, **p < 0.01, ***p < 0.001, Kruskal–Wallis one-way analysis of variance (ANOVA) test with Dunn’s comparison post-test.

We next assessed the frequency of circulating T peripheral helper (Tph) cells, defined as CD4^+^PD-1^+^CXCR5^–^, which accumulate in inflamed tissues and also have the ability to provide help to B cells^32,33^. We found that this population of cells is especially increased among SLE patients (*p* = 0.0054) (Fig. 3A), as previously described by other groups^34–36^. Although the Tph frequency among SLE patients follows a bimodal distribution, with a small subgroup of patients displaying higher levels of circulating Tph cells, we did not find an association with clinical parameters, such as disease duration, disease activity, autoantibody status, or treatment (Supplementary Fig. S6).

Finally, we assessed the frequency of circulating CD4^+^CXCR5^+^CD25^–^Foxp3^+^ cells. We called these cells CD25^–^ Tfr cells, given the similarities with the phenotype of *bona fide* Tfr cells. We found that circulating CD25^–^ Tfr cells were increased only in patients with SLE (*p* = 0.0096), with no differences observed for HT and RA (Fig. 3B). Given the exclusive association of this cell population with SLE, we performed a ROC analysis that identified the frequency of CD25^–^ Tfr cells as a significant predictor of SLE compared to HD (area under the curve (AUC): 0.744; *p* = 0.0009; Supplementary Fig. S7). Namely, values of CD25^–^ Tfr cells equal or above 0.61% (of total CD4^+^ T cells) have a sensitivity of 62.5% and a specificity of 71% for the diagnosis of SLE, in comparison with HD (Supplementary Fig. S7).

Further, SLE patients also had an increase in the total population of circulating CD25^–^ Treg cells (*p* = 0.0050) (Fig. 3B). This finding suggests that the increase of the CD25^–^ Tfr cells does not explain the greater frequency of total CD25^–^ Treg cells (independently of CXCR5 expression), which has been previously pointed out as a possible biomarker for the stratification of SLE patients regarding renal involvement^37^.

## Discussion

In recent years we witnessed major advances regarding the understanding of the regulation of humoral responses leading to antibody and autoantibody production. While Tfh cells promote GC formation, affinity maturation, and production of high-affinity antibodies, Tfr cells regulate the magnitude of the GC response and prevent autoimmunity in mice^23,38,39^. Even though it is tempting to assume that autoimmunity arises when the Tfr/Tfh balance is shifted by the reduction of Tfr cells or increased Tfh cells, there is evidence that human autoimmunity can coexist with a blood Tfr/Tfh ratio shifted in the opposite direction^14^. Several explanations may account for this apparently counter-intuitive finding. One possibility is that the frequency of T follicular cells in blood may not directly reflect their incidence in the secondary lymphoid organs or ELS. Studies showing T follicular cells in the affected organs in human autoimmunity are still rare, meaning that this correlation between blood and tissue has not been yet fully established^40,41^. Another possibility stems from the observation that chronic stimulation by autoantigens, and the subsequent humoral activity, can lead to persistent production of circulating Tfr cells (as it occurs following influenza vaccination)^4^.

However, there are several reports with inconsistent results, either for different diseases or focused on the same disease, as different methods are used to define Tfr and Tfh subsets. The same designation (Tfh or Tfr) has been used with cell populations with disparate phenotypic characteristics. For instance, Tfh cells are often considered as CD4^+^CXCR5^+^ cells, without exclusion of Foxp3^+^ Tfr cells^26,27^. These inconsistencies make it difficult to compare the different studies, thus not allowing to infer whether there is a uniform Tfr/Tfh dysregulation that explains the pathogenesis of humoral imbalance in human autoimmunity.

We studied a group of organ-specific and systemic autoimmune diseases, using a consistent analysis approach, to investigate whether common characteristics regarding the frequency of Tfr and Tfh subsets could be established. We found that there is a marked heterogeneity between different autoantibody-mediated autoimmune diseases regarding the frequency of circulating Tfr and Tfh subsets. It remains unclear whether the observed changes in T follicular cells are the cause or the consequence of the humoral immune dysregulation underlying these diseases. As a result, it is possible that some are actual autoantibody-mediated conditions, whereas others are diseases that have detectable autoantibodies, but are not necessarily mediated by them, thus contributing to the heterogeneity we observed. This could be overcome by accessing the dysregulation of the germinal center response directly in the affected tissues, both in the target organ and in draining lymphoid tissue where autoantibody production occurs. Unfortunately, in the present study we compared different diseases with distinct target tissues. Therefore, a tissue-based approach would risk increasing the heterogeneity of the analysis, further complicating the interpretation of our and previous findings.

Our findings support the hypothesis that T follicular cell dysregulation differs across autoimmune diseases, both organ-specific and systemic. However, we should underline that this study was not designed to identify blood biomarkers of specific autoimmune diseases, based on Tfh/Tfr cell patterns. Such a study would have limited applicability as HT, RA and SLE are very different diseases from a clinical point of view and, therefore, the use of a biomarker would likely not be clinically useful. However, there may be some potential for these initial data to be further explored within each disease, aiming at patient stratification or even personalized treatment. Indeed, the diverse patterns observed within a particular disease, raise the hypothesis that different subgroups of patients have distinct patterns of humoral dysregulation, which possibly also vary over time. It is well established that some clinical features, such as positivity for specific autoantibodies or the presence of ELS can occur in only a subgroup of patients with the same disease, such as in RA or pSS^14,42–44^.

This type of heterogeneity, linked to distinct immunopathogenesis, may provide an important explanation for different responses observed to the same treatment regimens. Thus, strategies to further stratify patients according to the underlying pattern of immune dysregulation, using Tfr/Tfh as a proxy, may contribute to develop tailored individualized treatments. In fact, we and others have previously shown that the frequency of circulating PD-1^+^ICOS^+^ Tfh cells could be used to identify pSS patients with greater disease activity^14,15^, whereas the Tfr/Tfh ratio could identify those patients with ELS within salivary glands^14^. This supports our hypothesis that, in specific diseases, subpopulations of Tfr and Tfh cells may allow patient stratification. In addition, other components of the inflammatory response, namely serum cytokine profiles, can potentially contribute to a better characterization of unique features of distinct autoimmune diseases and subgroups of patients with the same disease, providing additional tools for a more refined patient stratification.

Our study has several limitations. Patients with distinct diseases could not be fully matched for demographic or clinical characteristics, such as treatment or disease duration. Thus, it is possible that specific drugs or disease stage (i.e., early/late) may affect Tfr and Tfh subsets differently. Nevertheless, we were unable to find an association between the observed heterogeneity in Tfr and Tfh subsets and specific treatments or other clinical parameters, such as disease duration or serological markers. In addition, the number of patients included in each group may be relatively small, considering the described disease heterogeneity, especially in RA and SLE. Analyzing a larger number of patients with such conditions may overcome this issue, especially with regard to patient stratification, thus allowing for more robust conclusions. Also, the heterogeneity found in Tfr and Tfh subsets in patients under different treatments (Supplementary Fig. S8) reinforces the need for larger studies. It is also possible that the pattern of dysregulation in tissues with humoral responses driving autoantibody production may differ from the content of T follicular subsets in circulation. Nevertheless, the peripheral blood compartment shares common features across diseases, namely the presence of circulating autoantibodies and dysregulated humoral responses. In addition, it is easier to access and more thoroughly studied, with frequent claims of circulating Tfh/Tfr dysregulation as a universal hallmark autoantibody production—an assumption we have shown cannot be made.

In conclusion, humoral dysregulation in autoimmunity cannot be ascribed to a common mechanism related to T follicular cells. However, the heterogeneity of Tfr and Tfh subsets found across different autoimmune diseases, and within specific patient subgroups, offers an opportunity to explore potential biomarkers of the different diseases and for further studies on patient stratification. This may potentially contribute to developing tailored therapeutic strategies and improving patient outcomes.

## Supplementary Information

Supplementary Information.

## Data Availability

The data underlying this article will be shared on reasonable request to the corresponding author L.G.
