# Supplementary material for: Different antibody-associated autoimmune diseases have distinct patterns of T follicular cell dysregulation
Source: Sci Rep. 2022 Oct 21;12:17638. doi: 10.1038/s41598-022-21576-8 (PMC9587230; doi:10.1038/s41598-022-21576-8)
Supplement: Supplementary file 1 — Supplementary Information. [file 41598_2022_21576_MOESM1_ESM.pdf]

Supplementary Information

Different antibody-associated autoimmune diseases have distinct patterns of T follicular cell dysregulation

Filipa Ribeiro1,2[†], Vasco C. Romão1,3[†], Sara Rosa1, Kátia Jesus1, Ana Água-Doce1, Sofia C. Barreira1,3, Patrícia Martins1,3, Susana Lopes da Silva1,3, Ema Nobre3, Maria João Bugalho3, Válter R. Fonseca1*, João Eurico Fonseca1,3*, Luis Graca1,2*

[1] Instituto de Medicina Molecular João Lobo Antunes, Faculdade de Medicina da Universidade de Lisboa, Lisboa, Portugal
[2] Instituto Gulbenkian de Ciência, Oeiras, Portugal
[3] Hospital de Santa Maria, Centro Hospitalar Lisboa Norte, Lisboa, Portugal

[†] These authors contributed equally to this work.
***** Joint senior authors.

A

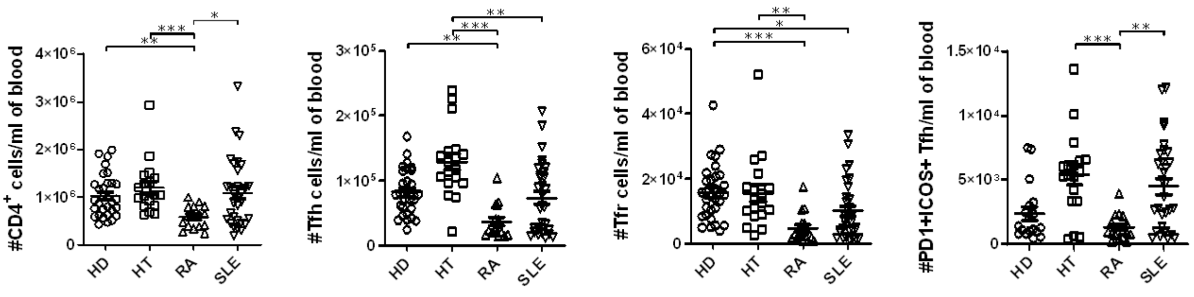

B

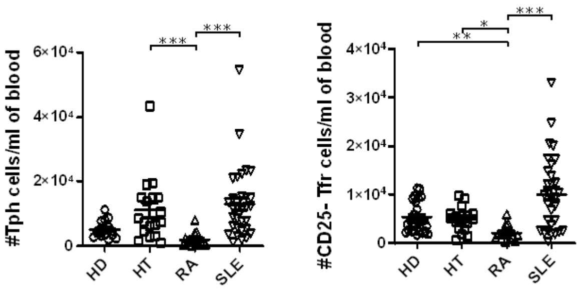

Supplementary Figure S1. Studied cell populations per volume of blood in the different diseases. (A) CD4$^+$ T cells, CD4$^+$CXCR5$^+$CD25$^-$Foxp3$^-$ Tfh cells, CD4$^+$CXCR5$^+$CD25$^+$Foxp3$^+$ Tfr cells, and activated PD-1$^+$ICOS$^+$ Tfh cells, **(B)** Tph cells and CXCR5$^+$CD25$^-$Foxp3$^+$ T cells per mL of blood of HT (n=18), RA, (n=16) and SLE (n=32) patients and HD controls (A: n=31; B: n=16). Each data point represents an individual subject; bars represent mean±SEM; * p<0.05, ** p<0.01, *** p<0.001, Kruskal-Wallis one-way analysis of variance (ANOVA) test with Dunn's comparison post-test.

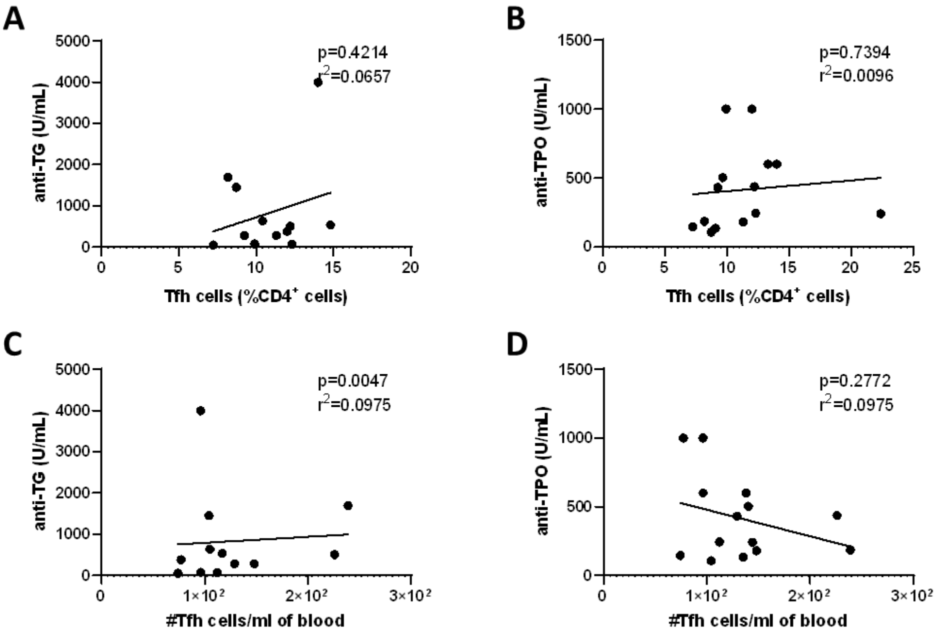

Supplementary Figure S2. Correlation between serum antibody titers and circulating Tfh cells in HT patients. Correlation of peripheral blood total Tfh cells, in percentage **(A,B)** and cell numbers **(C,D)**, with serum anti-TG (n=12; left) and anti-TPO titers (n=14; right) in HT patients. Linear regression is shown in all graphs (p = p-value; r^2 = coefficient of determination).

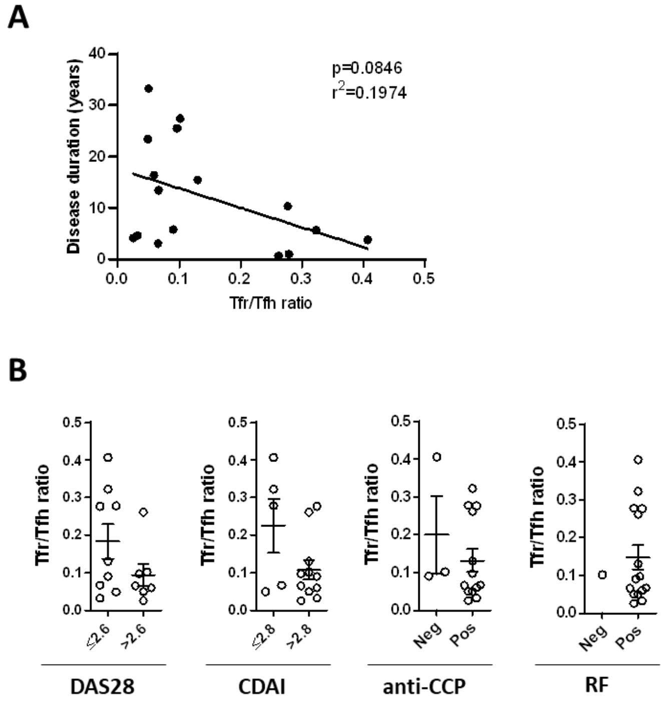

Supplementary Figure S3. Correlation between clinical parameters and circulating Tfr/Tfh ratio in RA patients. Heterogeneity of the Tfr/Tfh ratio in peripheral blood of RA patients (n=16) according to their clinical information: **(A)** disease duration and **(B)** Disease Activity Score-28 (DAS28), Clinical Disease Activity Index (CDAI), anti-cyclic citrullinated peptides (anti-CCP) and rheumatoid factor (RF). In **(A)** linear regression is shown. In **(B)**, symbols represent individual subjects; bars represent mean±SEM.

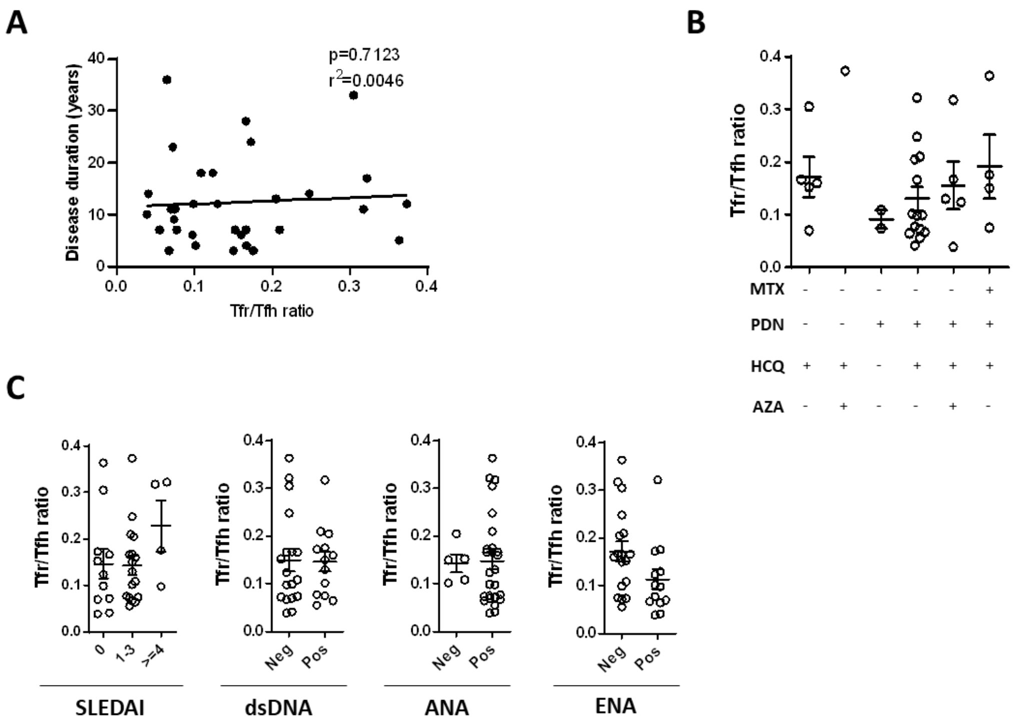

Supplementary Figure S4. Correlation between clinical parameters and circulating Tfr/Tfh ratio in SLE patients. Heterogeneity of the Tfr/Tfh ratio in peripheral blood of SLE patients (n=32) according to their clinical information: **(A)** disease duration, **(B)** treatments, and **(C)** Systemic Lupus Erythematosus Disease Activity Index (SLEDAI), anti-dsDNA antibodies, anti-nuclear antibodies (ANA) and extractable nuclear antigen antibodies (ENA). MTX = Methotrexate, PDN = Prednisolone, HCQ = Hydroxychloroquine, AZA = Azathioprine. In **(A)** linear regression is shown. In **(B)** and **(C)**, symbols represent individual subjects; bars represent mean±SEM.

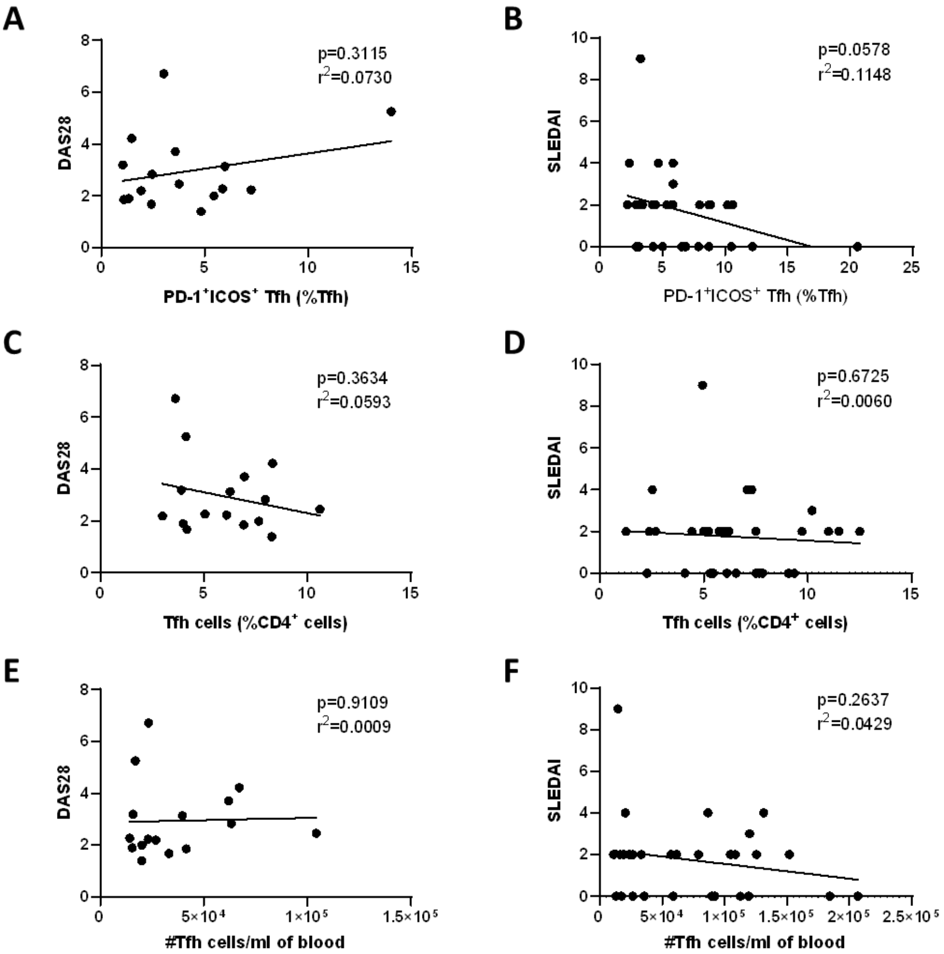

Supplementary Figure S5. Correlation between disease activity and circulating PD-1$^+$ICOS$^+$ and total Tfh cells in RA and SLE patients. Correlation of peripheral blood PD-1$^+$ICOS$^+$ Tfh cells **(A,B)** and total Tfh cells, in percentage **(C,D)** and cell numbers **(E,F)**, with DAS28 in RA patients (n=16; left) and SLEDAI in SLE patients (n=32; right). Linear regression is shown in all graphs (p = p-value; r^2 = coefficient of determination).

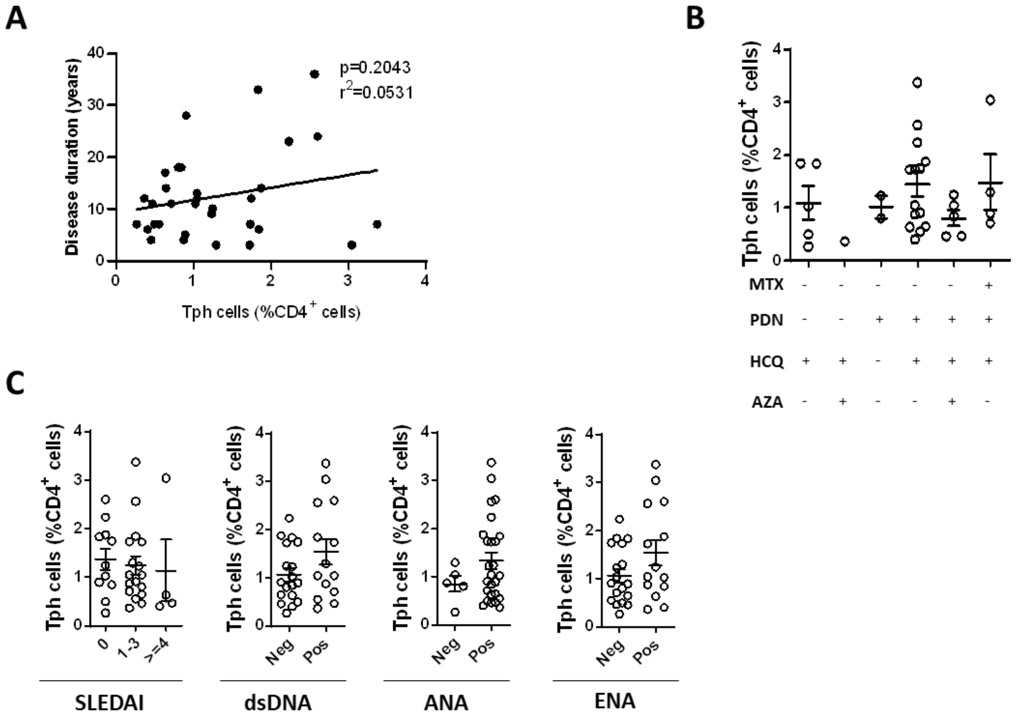

Supplementary Figure S6. Correlation between clinical parameters and circulating Tph cells in SLE patients. (A) Heterogeneity of the Tph cell population in peripheral blood of SLE patients (n=32) according to their clinical information: **(A)** disease duration, **(B)** treatments, and **(C)** Systemic Lupus Erythematosus Disease Activity Index (SLEDAI), anti-dsDNA antibodies, anti-nuclear antibodies (ANA) and extractable nuclear antigen antibodies (ENA). MTX = Methotrexate, PDN = Prednisolone, HCQ = Hydroxychloroquine, AZA = Azathioprine. In **(A)** linear regression is shown. In **(B)** and **(C)**, symbols represent individual subjects; bars represent mean±SEM.

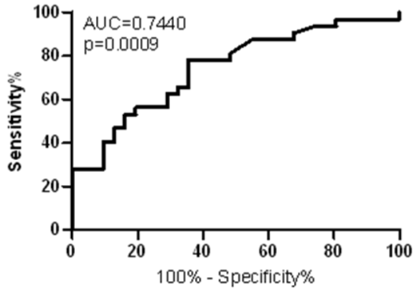

SLE vs HD	Sensitivity	Specificity	Likelihood ratio
CD25^{-} Tfr ≥ 0.61	62.50	70.97	2.153

Supplementary Figure S7. CD25^{-} Tfr cells as a biomarker of SLE. ROC curve for prediction of SLE diagnosis (SLE vs HD) based on CD25^{-} Tfr cells (AUC = area under the curve). Shown are percentages of sensitivity, specificity and likelihood ratio based on given cutoff.

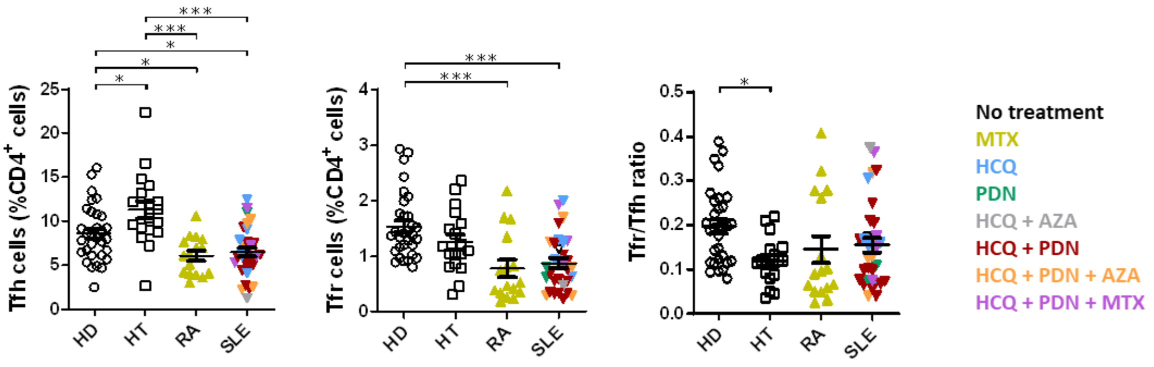

Supplementary Figure S8. Heterogeneity of circulating Tfh and Tfr cells of SS, RA, SLE, and HT and diversity of ongoing treatments. Frequency of $CD4^{+}CXCR5^{+}CD25^{-}Foxp3^{-}$ Tfh cells and $CD4^{+}CXCR5^{+}CD25^{+}Foxp3^{+}$ Tfr cells in peripheral blood of HD (n=31) and HT (n=18), RA (n=16) and SLE (n=32) patients (as described in Figure 1) colored by ongoing treatments. MTX = Methotrexate, HCQ = Hydroxychloroquine, PDN = Prednisolone, AZA = Azathioprine. All patients with HT are on levothyroxine treatment (not shown). Each data point represents an individual subject. Each data point represents individual subjects; bars represent mean±SEM. * $p<0.05$, ** $p<0.01$, *** $p<0.001$, Kruskal-Wallis test with Dunn's comparison post-test was applied.
